# Genome-wide identification and characterization of the *bHLH* gene family in tomato

**DOI:** 10.1186/s12864-014-1209-2

**Published:** 2015-01-22

**Authors:** Hua Sun, Hua-Jie Fan, Hong-Qing Ling

**Affiliations:** State Key Laboratory of Plant Cell and Chromosome Engineering, Institute of Genetics and Developmental Biology, Chinese Academy of Sciences, No. 1 West Beichen Road, Chaoyang District Beijing, 100101 China

**Keywords:** Tomato, *Solanum lycopersicum*, *bHLH* gene family, transcription factor, fruit development

## Abstract

**Background:**

The basic helix-loop-helix (bHLH) proteins are a large superfamily of transcription factors, and play a central role in a wide range of metabolic, physiological, and developmental processes in higher organisms. Tomato is an important vegetable crop, and its genome sequence has been published recently. However, the *bHLH* gene family of tomato has not been systematically identified and characterized yet.

**Results:**

In this study, we identified 159 bHLH protein-encoding genes (*SlbHLH*) in tomato genome and analyzed their structures. Although bHLH domains were conserved among the bHLH proteins between tomato and *Arabidopsis*, the intron sequences and distribution of tomato *bHLH* genes were extremely different compared with *Arabidopsis*. The gene duplication analysis showed that 58.5% and 6.3% of *SlbHLH* genes belonged to low-stringency and high-stringency duplication, respectively, indicating that the *SlbHLH* genes are mainly generated via short low-stringency region duplication in tomato. Subsequently, we classified the *SlbHLH* genes into 21 subfamilies by phylogenetic tree analysis, and predicted their possible functions by comparison with their homologous genes of *Arabidopsis*. Moreover, the expression profile analysis of *SlbHLH* genes from 10 different tissues showed that 21 *SlbHLH* genes exhibited tissue-specific expression. Further, we identified that 11 *SlbHLH* genes were associated with fruit development and ripening (eight of them associated with young fruit development and three with fruit ripening). The evolutionary analysis revealed that 92% *SlbHLH* genes might be evolved from ancestor(s) originated from early land plant, and 8% from algae.

**Conclusions:**

In this work, we systematically identified SlbHLHs by analyzing the tomato genome sequence using a set of bioinformatics approaches, and characterized their chromosomal distribution, gene structures, duplication, phylogenetic relationship and expression profiles, as well predicted their possible biological functions via comparative analysis with bHLHs of *Arabidopsis*. The results and information provide a good basis for further investigation of the biological functions and evolution of tomato bHLH genes.

**Electronic supplementary material:**

The online version of this article (doi:10.1186/s12864-014-1209-2) contains supplementary material, which is available to authorized users.

## Background

The basic helix–loop–helix (bHLH) proteins are a large superfamily of eukaryotic transcription factors, and play a central role in a wide range of metabolic, physiological, and developmental processes [1-3]. Their bHLH domain contains approximately 60 amino acids, including a basic region and a HLH region [4]. The basic region, which consists of approximately 17 amino acids and is located at the N-terminus of the domain, is a DNA-binding region that allows HLH proteins to bind to a consensus hexanucleotide E-box (CANNTG) [5,6]. The HLH region is composed of two amphipathic helices consisting of hydrophobic residues linked by a divergent (both in length and primary sequence) loop, and functions as a dimerization domain [4,7]. The HLH domain promotes protein–protein interactions and allows for the formation of homodimeric or heterodimeric complexes [6]. Excluding the conserved bHLH domain, the proteins showed considerable sequence divergence [5]. Furthermore, groups of evolutionary and/or functionally related bHLH proteins shared additional motifs. Some of these motifs have been characterized in animals regarding specificity in the DNA-binding sequence recognition and dimerization activities responsible for the activation or repression of target genes or for binding to small molecules [1].

Previous classifications of animal bHLHs have led to the definition of six major functional and evolutionary lineages (groups A–F) [1,8], which have been further subdivided into several smaller orthologous subfamilies [9]. Group A of the bHLH proteins can bind to the E-box sequence. In group B, several proteins such as Max, Myc, MITF, SREBP and USF have diverse functions and bind to the G-box sequence CACGTG [10-12]. Members of Group C contain an additional protein–protein interaction region and bind to sequences (NACGTG or NGCGTG) that do not resemble the E-box. Proteins in group D contain only the HLH region and can form heterodimers with bHLH proteins and thus are functionally related to typical bHLH proteins [13]. Group E proteins contain Pro or Gly residues within the basic region and can bind preferentially to the CACGNG sequence [14,15]. Group F proteins contain divergent sequences compared with other groups and another domain for dimerization and DNA binding [16].

Studies on the *bHLH* gene family in various species will increase our understanding of their evolution and functions. However, systematic identification of the *bHLH* genes has been performed only in a few plants, such as *Arabidopsis*, rice, poplar, and moss [2,17-19]. Tomato (*Solanum lycopersicum*) is one of the most important vegetables in the world and is also a model plant for studying fruit development [20]. Tomato genome sequencing was recently completed and published [21]. But the *bHLH* gene family of tomato has still not been reported. In this study, we systematically identified and characterized the *bHLH* genes of tomato (*SlbHLH*) and compared them with the *bHLH*s of *Arabidopsis thaliana*. In addition, we also analyzed the expression profiles of *SlbHLH* genes in different tissues and at different stages of fruit development as well in response to iron-deficiency stress. Finally, we detected several genes associated with fruit development and ripening, and with iron-deficiency responses. In addition, we did the evolutionary analysis of *SlbHLH* genes by comparison of tomato *bHLH* genes with that of angiosperm, early land plants and algaes.

## Results and discussion

### Identification and classification of tomato *bHLH* genes

For genome-wide identification of *bHLH* genes in tomato, we initially identified the proteins using two approaches described in the Methods and filtered the results based on the criteria developed by Atchley *et al*. [5] and Toledo-Ortiz *et al.* [2]. The bHLH domain contained 19 conserved amino acid residues, including five in the basic region, five in the first helix, one in the loop, and eight in the second helix [5] (Table 1). Based on the criterion for identifying *Arabidopsis* bHLH proteins (AtbHLHs) [2], we allowed nine mismatches from the 19 conserved amino acid residues for the identification of tomato bHLHs. To further confirm and filter uncertain bHLH proteins, we performed SMART analysis and retained proteins with e-values less than 1 (the e-value setting based on testing AtbHLH family). Finally, we identified 159 genes encoding bHLH proteins (SlbHLHs) in the tomato genome (Table 1 and Additional file 1) and named them according to the system proposed for *Arabidopsis* [17] (Additional file 1). Compared with the recent report by The Tomato Genome Consortium [21], 57 bHLH proteins were absent in this study because they did not conform to our minimal criteria for inclusion. These proteins contained an incomplete bHLH domain, has a low similarity (e-value ≥ 0.01) with the bHLH domain profile, or more than nine mismatches within the 19 conserved amino acid residues.

**Table 1:** **bHLH domain consensus motif**

	**Atchley et al. [** 5 **]**		**Toledo-Ortiz et al. [** 2 **]**		**This study**	
	**Position in the alignment**	**Consensus motif amino acid frequency within the bHLH domain**	**Position in the alignment**	**Amino acid frequency within the Arabidopsis bHLH domains**	**Position in the alignment**	**Amino acid frequency within the tomato bHLH domains**
Basic	1	R (61%), K (27%)	1	R (24%), K (22%)	1	K(28%), R(25%), N(11%)
	2	R (77%), K (16%)	2	R (35%)	2	R(32%), K(11%)
	9	E (93%)	13	E (76%), A (10%)	13	E(75%), A(11%)
	10	R (81%), K (14%)	14	R (74%), K (14%)	14	R(76%), K(18%)
	12	R (91%)	16	R (91%)	16	R(94%)
Helix	16	I (35%), L (33%), V (23%)	20	I (52%), L (27%), M (12%)	20	I(53%), L(28%), M(17%)
	17	N (74%)	21	N (51%), S (19%)	21	N(45%), S(26%)
	20	F (72%), L (14%), I (9%)	24	F (26%), L (26%), M (20%), I (14%)	24	L(28%), F(26%), M(19%), I(16%)
	23	L (98%)	27	L (100%)	27	L(99%)
	24	R (44%), K (35%)	28	Q (42%), R (35%)	28	Q(41%), R(37%)
Loop	47	K (58%), R (24%)	39	K (66%)	36	K(68%)
Helix	50	K (93%)	42	K (45%), T (13%)	47	K(45%), T(21%)
	53	I (74%), T (15%), V(7%)	45	M (33%), I (27%), V (16%), L (14%)	50	M(33%), I(28%), V(15%), L(14%)
	54	L (98%)	46	L (76%), V (14%)	51	L(78%), I(11%)
	57	A (76%)	49	A (60%), I (16%), V (12%)	54	A(60%), I(18%), V(11%), T(10%)
	58	I (31%), V (27%), T (23%)	50	I (63%), V (22%)	55	I(60%), V(25%)
	60	Y (77%)	52	Y (78%)	57	Y(74%), H(13%)
	61	I (69%), L (16%), V (8%)	53	I (40%), V (33%), L (13%)	58	I(43%), V(38%), L(13%)
	64	L (80%), M (7%)	56	L (93%)	61	L(97%)

**Figure 1:**
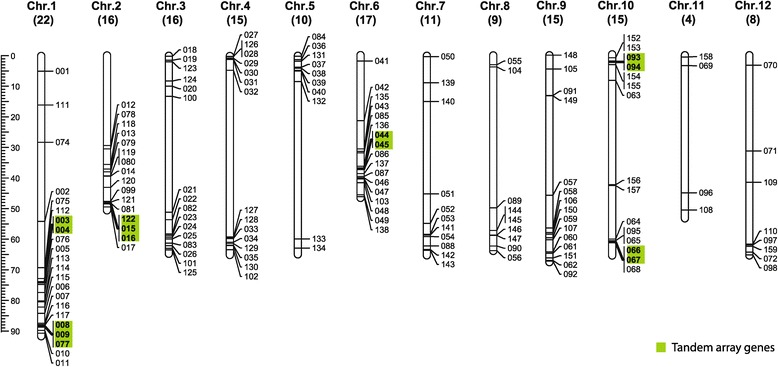
**Chromosomal localizations and tandem array of** ***SlbHLH*** **genes.** The numbers below the chromosome names show the total number of *SlbHLH* genes located on each chromosome. The green block indicates tandem array genes.

Based on information of gene annotation [21], 158 of the predicted 159 *SlbHLH* genes were localized on tomato chromosomes, while *SlbHLH073* was failed to locate on the 12 chromosomes [21]. As shown in Figure 1, the congregate region and the number of *SlbHLH*s are irregular although the *SlbHLH* genes distributed on all of the 12 tomato chromosomes. Chromosome 1 contained the largest number (22) of *bHLH* genes, whereas only four *bHLH* genes were present on chromosome 11. Based on the gene number of each chromosome, the percentage of *bHLH* genes per chromosome varied from 0.17% on chromosome 11 to 0.6% on chromosomes 6 and 9 with an average ratio of 0.41% (Additional file 2). According to similarity of SlbHLH proteins, we speculated that 103 genes were generated by gene duplication. Of them, 93 *SlbHLHs* belonged to the low-stringency duplication, and 10 to the high-stringency duplication (Additional file 3). Based on chromosome localization, 14 of 159 *SlbHLHs* were tandem array genes (TAGs) (Figure 1). The segment duplication analysis showed that *SlbHLH149* and *Solyc01g014960* were generated via a segment duplication (Additional file 4, Additional file 5A, B). However, *Solyc01g014960* did not contain a complete bHLH domain, suggesting it was a pseudogene. Based on our analysis, we propose that the majority of *SlbHLHs* may have been generated via gene duplication rather than segment duplication in the genome of tomato.

The tomato ‘Heinz 1706’ genome, with a genome size of approximately 900 Mb, was 7.2 times larger than the *A. thaliana* genome. However, the number of *SlbHLH* genes was similar to *Arabidopsis* (162). Based on the total genes, the ratio to *SlbHLH* genes in the tomato genome was about 0.46%, which was similar to rice (0.44%) and poplar (0.40%) [18,19], but was less than *Arabidopsis* (0.59%) [2,17].

### Conserved amino acid residues in the bHLH domains and DNA-binding ability

**Figure 2:**
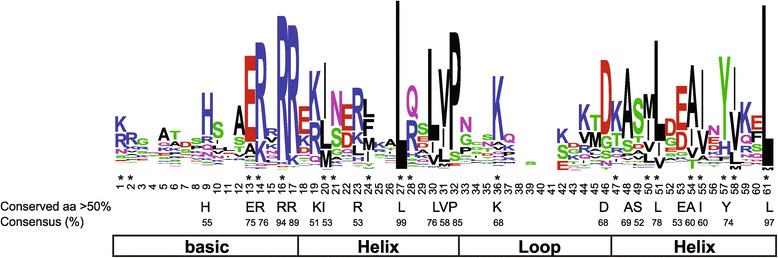
**The bHLH domain is highly conserved across all SlbHLH proteins.** The overall height of each stack represents the conservation of the sequence at that position. The asterisk indicates the position of the 19 conserved amino acids previously identified by Atchley *et al*. [5], and capital letters indicate over 50% conservation of amino acids among the 159 SlbHLH domains.

The bHLH domain alignment of 159 SlbHLHs showed that 22 amino acid residues in their bHLH domains were conserved with more than 50% consensus ratio (Figure 2). Compared with bHLHs of animals that described by Atchley et al. [5], we found that the residues Ile-20, Leu-24, Gln-28, Lys-36, Met-50, Ile-55, Val-58 and Leu-61 in the bHLH domains of plants were more conserved than in animals, suggesting that the seven amino acid residues may play an important role in plants (Table 1). Further, the residues Arg-16, Leu-27, and Leu-61 showed extreme conservation among the 159 bHLH proteins of tomato. Previous reports revealed that Glu-13 and Arg-16/Arg-17 in the basic region of bHLH domain play an important role in DNA binding [8], and Leu-27 and Leu-61 in the helix regions function in dimerization activity [19].

The basic region of the bHLH proteins contained 17 amino acids in tomato, which is four amino acids longer than that described by Atchley *et al*. [5] (Figure 2 and Table 1). The DNA-binding activity of target genes is determined in the basic region of the bHLH domain [6]. Based on the criteria developed by Toledo-Ortiz *et al*. [2], we defined proteins with more than five basic amino acid residues in the basic region as DNA-binding proteins. Furthermore, the DNA-binding proteins were subdivided into two subcategories, E-box (including G-box binder) and non-E-box binders (Table 2 and Additional file 1). Glu-13 and Arg-16 are known to be essential for recognition of the E-box, and His/Lys-9, Glu-13, and Arg-17 are required for binding of G-box [7,22]. According to the conservation of the residues, we predicted 98 SlbHLHs as putative E-box-binding proteins, and 12 as non-E-box-binding proteins (due to lacking Glu-13/Arg-16 residues). Among the 98 E-box-binding proteins, 72 belonged to G-box-binding proteins, while 26 proteins lacked the G-box-binding site (Table 2). Additionally, 49 of 159 SlbHLHs contain less than six amino acid residues in the basic region, and were classified as non-DNA-binding proteins (Table 2).

**Table 2:** **Predicted DNA-binding categories based on the bHLH domain**

**Predicted activity**	**Predicted motif**	**Number of AtbHLHs**	**Number of SlbHLHs**
		**(Toledo-Ortiz et al. [** 2 **])**	**(This study)**
DNA binding			
E-box			
G-box	bHLH	89 (60.54%)	72 (45.28%)
Non-G-box	bHLH	20 (13.61%)	26 (16.35%)
Non-E-box	bHLH	11 (7.48%)	12 (7.55%)
Total		120 (81.63%)	110 (69.18%)
Non-DNA binding	HLH	27 (18.37%)	49 (30.82%)

### Intron distribution and phylogenetic analysis of *SlbHLH* genes

**Figure 3:**
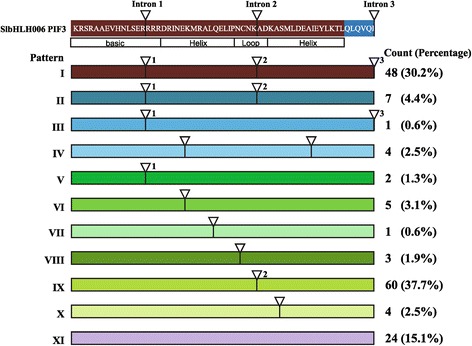
**Intron distribution patterns in the coding sequence of the bHLH domain of** ***SlbHLHs.*** Scheme of the intron distribution patterns (color coded and designated I to XI) within the bHLH domain of *SlbHLHs*. Position of introns is indicated by triangles and numbered (1 to 3) based on the bHLH region of PIF3, which is shown at the top. When the position of the intron coincides with that found in PIF3, the intron number is given above the triangle. The count and percentage of genes in each pattern are given on the right.

To analyze intron distribution within the coding sequence of the bHLH domain in all *SlbHLH* genes, we did alignment analysis with the coding and genome sequences using Blat software, and found 11 different intron distribution patterns (designated as I to XI). The intron number ranged from 0 to 3 within the coding sequence of the bHLH domain (Figure 3 and Additional file 1). As shown in Additional file 6, 85% of *SlbHLH*s contained introns in their coding sequence of the bHLH domain, and 74% had a conserved intron position, but the sequence length and similarity of the introns were quite different, even at the same position. In contrast, 15% of the *SlbHLH*s did not contain intron in their bHLH domain region (pattern XI). Pattern I (including three introns) was the most common pattern in *Arabidopsis* [2], but it was only the second common one in tomato. These results showed that intron sequences and distribution between tomato and *Arabidopsis* were different although their bHLH domains were conserved.

**Figure 4:**
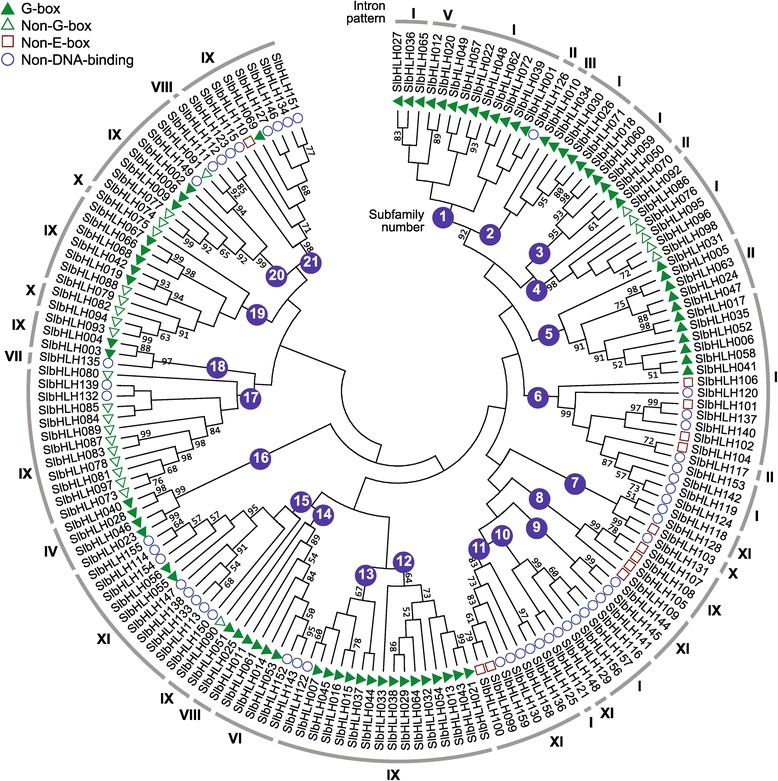
**Relationship of neighbor-joining phylogenetic tree of the SlbHLH domains with the predicted DNA-binding activities and the intron distribution patterns.** The tree shows the 21 phylogenetic subfamilies marked with white font on a colored background. Roman numerals correspond to the intron patterns shown in Figure 3. The different shape on the left side of SlbHLH represents the predicted DNA-binding activity of each protein.

To explore whether the intron distribution pattern and DNA-binding activity correlate with their phylogenetic classification, a neighbor-joining phylogenetic tree was built using alignments of bHLH domains. The phylogenetic tree showed that 159 bHLH domains were classified into 21 subfamilies (Figure 4). The genes with E-box binding region was mostly clustered within the subfamilies 1–5, 12–14, and 16–20, whereas the genes with non-DNA-binding region were grouped in the subfamilies 6–11 and 21. Moreover, intron distribution pattern I (corresponding to the subfamily 6) and pattern IX (corresponding to the subfamilies 8, 12, 13, and 17–21) mainly belonged to the E-box-binding type, and pattern XI (corresponding to the subfamilies 7, 9, 11 and 15) to non-DNA-binding type. These results suggest that some rules and dependencies exist between the intron distribution pattern and DNA-binding activity among the SlbHLHs.

Up to now, the biological functions of the most SlbHLHs remain unclear. However, approximately 40% of Arabidopsis bHLH proteins have been functionally characterized. Hence, the clustering and comparison of tomato bHLH proteins with AtbHLHs can help to predict their functions via ortholog analysis. The phylogenetic analysis revealed that 43 SlbHLHs were tightly grouped with the AtbHLHs, in which their functions are known. These suggest that the 43 SlbHLH proteins may have the similar functions as their *Arabidopsis* ortholog (Additional file 7 and Additional file 8).

### Expression pattern of *SlbHLH* genes among different tissues

To analyze the expression pattern of *SlbHLH* genes among ten different tissues, we used the Reads Per Kilobase per Million (RPKM) normalized data from RNA-seq [21]. Additional file 9 showed the expression profiles of *SlbHLH* genes in the ten tomato tissues. Among 159 *SlbHLHs* genes, 122 expressed at least in one of the ten tissues with an RPKM value greater than 1.4, while the rest 37 exhibited a low expression (RPKM ≤ 1.4) in all ten tissues. Moreover, we found that 21 *SlbHLHs* displayed the tissue-specific expression preference (the expression intensity was more than 2 times higher in a particular tissue than that of other tissues). They include eleven genes (*SlbHLH034, SlbHLH038, SlbHLH050, SlbHLH053, SlbHLH057, SlbHLH085, SlbHLH092, SlbHLH105, SlbHLH120, SlbHLH128, SlbHLH137*) in root, three genes (*SlbHLH024, SlbHLH041, SlbHLH094*) in leaf, three genes (*SlbHLH001, SlbHLH022, SlHLH059*) in flower, one gene (*SlbHLH089*) in bud, and three genes (*SlbHLH006, SlbHLH078, SlbHLH095*) in fruit (Additional file 9), implying that they may have some functions in these tissues, respectively.

### Fruit-development-related *SlbHLH* genes and their *cis*-element analysis

**Figure 5:**
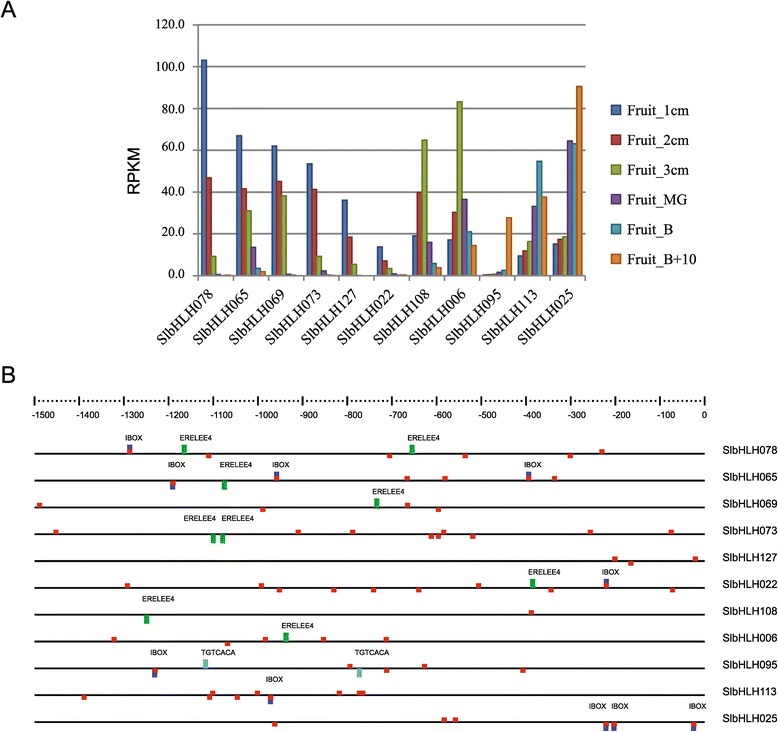
**Expression patterns of the** ***SlbHLH*** **genes related fruit development and their** ***cis*** **-element analysis. (A)** The expression profiles of the indicated genes at different stages of fruit development and ripening. The expression data of the *SlbHLH* genes were obtained from the RNA-seq data of Solanaceae Genomics Network (http://solgenomics.net; ITAG Release 2.3). Fruit_1cm, Fruit_2cm and Fruit_3cm mean the RNAs extracted from 1 cm, 2 cm and 3 cm large fruits, respectively. Fruit_MG indicates mature green (MG), Fruit_B is breaker (early ripening), and Fruit_B + 10 means 10 days post-B (red ripe). **(B)** *Cis*-element analysis of the indicated genes from upstream 1500 bp sequence to the transcription start site. The upside represents the forward sequence element and downside represents the matching element with the reverse. Red-, blue-, green-, and light blue-colored blocks mean I-box core-, I-box-, Erelee4-, and TGTCACA-elements, respectively.

Tomato is a model plant for studying fruit development and ripening. Based on gene expression data, we identified 11 *SlbHLH* genes which showed a gradually increased or decreased expression with fruit development and ripening. Of them, 6 genes (*SlbHLH022*, *SlbHLH065*, *SlbHLH069*, *SlbHLH073*, *SlbHLH078*, and *SlbHLH127*) exhibited a high expression in 1-cm large fruit, and two (*SlbHLH006* and *SlbHLH108*) in 3-cm large fruit in comparison with other tissues (Figure 5A). These data indicate that the eight *SlbHLH* genes may be involved in fruit development. The expression of the rest three genes (*SlbHLH025*, *SlbHLH095*, and *SlbHLH113*) was gradually upregulated during the ripening process, suggesting that they may function in fruit ripening. Indeed, it was reported recently that *SlbHLH095* (*Solyc10g079050*) was associated with fruit ripening [23]. Therefore, further characterization of the 11 *SlbHLHs* is highly important and will provide a new insight to understand the molecular mechanism of fruit development and ripening.

To investigate the regulation mechanisms of the 11 fruit-related *SlbHLH* genes, we analyzed their *cis*-elements from the transcriptional start site to the –1500-bp upstream region (Figure 5B). In the 11 *SlbHLHs*, 172 elements were detected using PLACE (http://www.dna.affrc.go.jp/PLACE/), of which 22 were mutual elements. Ethylene is known to be important for fruit development and ripening [20]. Thus, we examined ethylene-regulated elements using bioinformatics tool. Ethylene-responsive elements (ERELEE4, AWTTCAAA) were found in seven genes (*SlbHLH006*, *SlbHLH022*, *SlbHLH065*, *SlbHLH069*, *SlbHLH073*, *SlbHLH078*, and *SlbHLH108*), which were involved in young fruit development, but were not detected in the three *SlbHLH* genes related to fruits ripening (*SlbHLH025*, *SlbHLH095*, and *SlbHLH113*). In previous study, the TGTCACA motif was shown to be an enhancer element required for fruit-specific expression of the *cucumisin* gene from melon, and the I-box-like sequence AGATATGATAAAA functions as a negative regulatory element [24]. Among the fruit-specific *bHLH* genes of tomato, only *SlbHLH095* contained the TGTCACA element in promoter. Although the I-box-like motif was not detected in the promoter region of the 11 *SlbHLH*s, the I-box core element (GATAA) and I-box (GATAAG) presented commonly. Considering the distribution of *cis*-elements in the promoter of these genes, we speculate that they may play some roles in regulating the expression of the corresponding genes for the fruit development and ripening.

### Prediction of SlbHLHs involved in the regulation of iron deficiency responses and homeostasis

**Figure 6:**
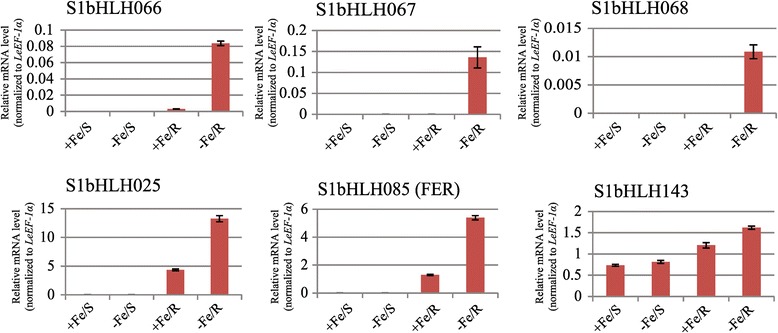
**Expression analyses of the six** ***SlbHLH*** **genes under iron-deficient stress by qRT-PCR.** For qRT-PCR, the relative amount of mRNA (y-axis) was calculated by according to the description in Methods. The x-axis indicates the shoot (S) and root (R) of tomato under iron-sufficient (+Fe) and iron deficient (-Fe) conditions.

Iron deficiency is one of mostly limiting factors for plant growth and development. Its uptake and homeostasis are tightly regulated because excess and deficiency of iron are pernicious for plants. Several bHLH transcription factors involved in the regulation of iron uptake and homeostasis have been cloned and characterized in *Arabidopsis*. They are the FER-like iron-deficiency-induced transcription factor (FIT), bHLH38, bHLH39, bHLH100, bHLH101, POPEYE (PYE), and IAA–Leu Resistant3 (ILR3) [25-28]. In tomato, only one bHLH transcriptional regulator (FER), which is involved in regulating iron-deficiency responses and uptake, has been characterized [29]. In *Arabidopsis*, FIT is an ortholog of tomato FER [25]. Under iron deficiency, FIT interacts with bHLH38, bHLH39, bHLH100 or bHLH101 to form heterodimer(s) and activate the expression of their downstream genes, such as *FRO2* and *IRT1* [26,28]. PYE interacts with ILR3, functioning in the negative regulation of iron deficiency responses [27]. To predict SlbHLHs which may function in the responses to iron deficiency and homeostasis, we compared the tomato bHLHs with AtbHLHs. As shown in Additional file 7, six SlbHLHs (SlbHLH025, SlbHLH066, SlbHLH067, SlbHLH068, SlbHLH085, SlbHLH143) were correspondingly clustered with the 7 AtbHLHs (FIT, AtbHLH38, AtbHLH39, AtbHLH100, AtbHLH101, PYE, ILR3), which are involved in iron deficiency responses and homeostasis. SlbHLH085 is FER and the ortholog of FIT (AtbHLH29) [25]. SlbHLH025 is corresponded to AtbHLH047 (PYE), SlbHLH143 to AtbHLH105 (ILR3), whereas SlbHLH066, SlbHLH067 and SlbHLH068 were grouped with AtbHLH38, AtbHLH39, AtbHLH100 and AtbHLH101 together. Further, we analyzed the expression profiles of the six *SlbHLHs* in shoots and roots using qRT-PCR. The genes *SlbHLH066*, *SlbHLH067* and *SlbHLH068*, which are corresponding to *AtbHLH38*, *AtbHLH39*, *AtbHLH100* and *AtbHLH101* of *Arabidopsis*, expressed specifically in roots under iron-deficient conditions (Figure 6), whereas the four *Arabidopsis* genes expressed in roots and also in leaves [26,28]. These results imply that the transcriptional regulation of these *bHLH* genes has some differences between tomato and *Arabidopsis*. In addition, *SlbHLH025* (ortholog of *PYE*) and *SlbHLH085* (*FER*) were also specifically expressed in root, and were highly expressed under iron deficiency. However, the expression of *SlbHLH143* (corresponding to *Arabidopsis ILR3*) was not distinct between iron deficiency and sufficiency in shoots while its expression was upregulated only in roots under iron deficiency (Figure 4). All these suggest that SlbHLH066, SlbHLH067 and SlbHLH068 may have similar function as AtbHLH38, AtbHLH39, AtbHLH100 and AtbHLH101 in the responses to iron deficiency and uptake, and that SlbHLH025, like PYE of *Arabidopsis*, may function in the negative regulation of iron deficiency responses. However, more experiments are needed to confirm it.

### Evolutionary relationship of *SlbHLH* genes

Plant kingdom is divided into algae (including red algae, chlorophyta) and land plant (mosses, lycophytes, and angiosperms) [30]. Its evolution is from algae to land plant. Recently, Pires and Dolan [31] defined the relationships of bHLH proteins in plant kingdom using the whole-genome sequences of nine species from algae and land plants. They showed that only few (less than 5) bHLH proteins were detected in the genomes of chlorophytes and red algae. In contrast, many bHLH proteins (100–170) are identified in the genomes of land plants (embryophytes). Phylogenetic analyses suggest that plant bHLH proteins are monophyletic and much of the bHLH protein diversity in plant kingdom was established in early land plants, over 440 million years ago [31].

**Figure 7:**
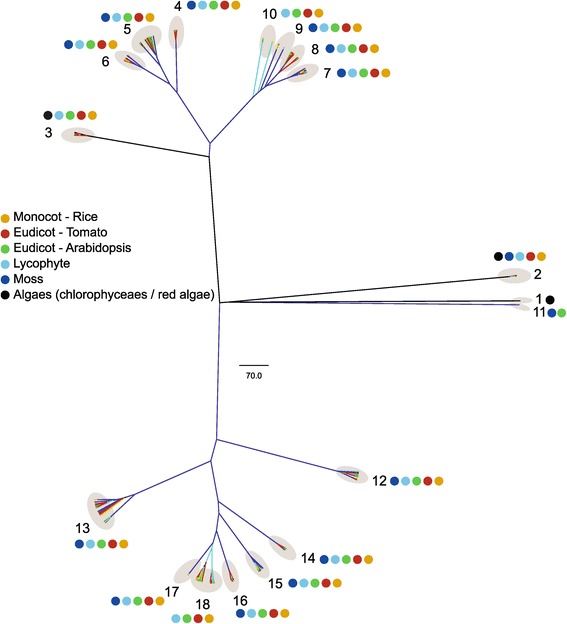
**Phylogenetic relationships in the vascular plants, moss, and algaes.** Maximum likelihood analysis of 696 plant bHLHs show as cladogram, and was rooted with a node of red algae protein. The gray balloons delineate the 18 subfamilies of bHLH proteins. Colored dots symbolize the species to which the bHLH proteins in each group belong (yellow: *Oryza sativa* [monocot]; red: *Solanum lycopersicum* [eudicot]; green: *Arabidopsis thaliana* [eudicot]; light-blue: *Selaginella moellendorffii* [lycophyte]; purple: *Physcomitrella patens* [moss]; black: *Volvox carteri*, *Chlamydomonas reinhardtii*, and *Cyanidioschyzon merolae* [chlorophytes and red algae]). A full tree with protein names is given in Additional file 10.

To observe the evolutionary relationship of tomato *bHLH* genes, SlbHLHs were compared with bHLH protein data of other plants described by Pires and Dolan [31]. The phylogenetic tree was constructed with the bHLH domain regions of bHLH proteins of 8 species (1 monocot, 2 eudicots, 1 lycophyte, 1 moss, 2 chlorophyceaes, and 1 red algae). As shown in Figure 7 and Additional file 10, total 696 bHLH proteins of the 8 species were classified into 18 subfamilies based on clade and evolution of species in the topology of the trees. The 159 bHLHs of tomato were distributed into 16 of 18 subfamilies except of the subfamily 1 and 11. Based on the subfamily classification, 13 SlbHLHs were clustered together with algae bHLHs in the subfamily 2 and 3, 127 with moss bHLHs in 12 subfamilies (subfamily 4-9, 12-17), and 19 with lycophyte bHLHs in the subfamily 10 and 18. These results suggest that the 13, 127 and 19 SlbHLHs may evolve from ancestors of algae, moss and lycophyte, respectively. The oldest evidence for the existence of vascular plants comes from trilete spores found in Upper Ordovician sediments, over 443 Ma [32], and green algae chlorophyceae from 1 billion years ago [33]. Hence, we speculate that 92% of SlbHLH proteins should be evolved from ancestor(s) originated in land plants over 443 Ma, and 8% from the ancestor(s) appeared in algae over 1 billion years ago. These are consistent with the report by Pires and Dolan [31].

## Conclusions

In this work, we systematically analyzed the sequence of tomato genome, identified 159 bHLH genes, and characterized their structure, duplication, chromosomal distribution, phylogenetic tree, and expression patterns. Among the 159 *SlbHLHs*, the expression of 11 and 6 *SlbHLHs* was related to fruit development and ripening, and to response of iron deficiency, respectively. Further, we annotated the possible biological functions of 43 SlbHLHs by comparative analysis with bHLHs of *Arabidopsis*. The evolution analysis showed that all of SlbHLHs are highly conserved in plant evolution and much of the diversity of SlbHLH proteins was established in early land plants. Taken together, the results and information described in this work provide a good basis for further investigation of the biological functions and evolution of tomato *bHLH* genes.

## Methods

### Data set collection and identification of *bHLH* genes

Tomato genome sequence data were obtained from the Solanaceae Genomics Network (SGN) in May 2012 (http://solgenomics.net; ITAG Release 2.3) [21]. The information and sequences of *A. thaliana* bHLHs (AtbHLHs) were retrieved from The Arabidopsis Information Resource (TAIR; http://www.arabidopsis.org/), *Oryza sativa* bHLHs (OsbHLHs) were obtained from Li et al. [18], and a data set of bHLH proteins from early land plant (Lycophyte - *Selaginella moellendorffii*, and Moss - *Physcomitrella patens*) and algaes (Chlorophyceae - *Volvox carteri* and *Chlamydomonas reinhardtii*, and Red algae - *Cyanidioschyzon merolae*) was retrieved from Pires and Dolan [31]. The bHLH proteins of tomato (SlbHLHs) were predicted using the HLH hidden Markov model (HMM) profile obtained from Pfam (http://pfam.xfam.org, PF00010) and used as queries to search the bHLH proteins from tomato sequences with HMMER software (http://hmmer.janelia.org). In addition, the previously known AtbHLH sequences were applied as input to build a bHLH consensus domain profile with MEME software (http://meme.nbcr.net/meme/). The profile was then used as queries to identify bHLH proteins using the MAST program (http://meme.nbcr.net/meme/) with tomato sequences. To further confirm and filter uncertain bHLH proteins, the predicted bHLH domains were examined with the SMART tool (http://smart.embl-heidelberg.de). The all of the bHLH protein sequences used in this study were showed in Additional file 11.

### Alignment and phylogenetic analysis

Multiple domain alignments were performed using Multalin (http://multalin.toulouse.inra.fr) and Clustal-omega tool (v 1.2, http://www.clustal.org). To visualize the conserved motifs, the sequences were analyzed with WEBLOGO programs (http://weblogo.berkeley.edu). Phylogenetic tree was constructed using MEGA5 (http://www.megasoftware.net) with the neighbor-joining method and the following parameters: pairwise deletion option, 1000 replicates of bootstrap and Poisson correction distance. The consensus tree showed only branches with a bootstrap consensus > 50. The maximum likelihood (ML) analyses were done with the program PhyML version 3.0 (http://www.atgc-montpellier.fr/phyml) using the JTT model of amino acid substitution, the radial tree was drawn using FigTree v1.3.1 (http://tree.bio.ed.ac.uk/software/figtree).

### Gene duplication pattern and location of *SlbHLH* genes on chromosomes

Gene duplication was classified into two groups: low-stringency duplication (protein pairs with ≥ 30% identity and covering ≥ 70% protein length) and high-stringency duplication (protein pairs with ≥ 50% identity and covering ≥ 90% protein length) [34]. TAGs were defined if they belonged to the same superfamily and were either physically adjacent or separated by a specific number of nonhomologous intervening “spacer” genes [34]. Segmental duplications (length ≥ 3 kb; identity ≥ 90%) were identified using MUMmer (http://sourceforge.net/projects/mummer) in the whole genome sequences of tomato. The *SlbHLH* genes were mapped onto the corresponding chromosomes by identifying their chromosomal positions provided in the SGN. The distribution of *SlbHLH* genes on chromosomes were drawn using MapChart software (http://www.wageningenur.nl/en/show/Mapchart.htm).

### Gene expression analysis and *cis*-element prediction

The expression pattern of the genes in different tissues was drawn using R script based on an average of normalized expression (RPKM mapped reads) of tomato *bHLH* genes from RNA-seq data [21]. The *cis*-element was predicted by PLACE (http://www.dna.affrc.go.jp/PLACE/).

### qRT-PCR analysis

After germination for 5 days, the tomato seedlings (Heinz) were cultured in a modified half-strength Hoagland nutrient solution [35] for 4 days. The nutrient solution contained 3.0 mM KNO_3_, 2.0 mM Ca(NO_3_)_2_·4H_2_O, 1.0 mM NH_4_H_2_PO_4_, 0.5 mM MgSO_4_·7H_2_O, 1.0 μM KCl, 25.0 μM H_3_BO_3_, 2.0 μM MnSO_4_·4H_2_O, 2.0 μM ZnSO_4_·7H_2_O, 0.1 μM CuSO_4_·5H_2_O, 0.1 μM (NH_4_)_6_Mo_7_O_24_·4H_2_O, 2.0 mM MES, and 20.0 μM Fe(Na)–EDTA, and the pH of the solution was adjusted to 5.5 with KOH. The 5-days-old seedlings were transferred into the one-half strength modified Hoagland solution with or without iron supply, and cultivated for 3 days. Subsequently, the roots and shoots were separately harvested, and their total RNAs were extracted. After eliminating genomic DNA contamination by DNase I (Fermentas, Waltham, MA, USA), about 2.0 μg of total RNA were used for the synthesis of first-strand complementary DNA (cDNA) with the cDNA Synthesis Kit (Invitrogen, Carlsbad, CA, USA) and subjected to qRT-PCR analysis using the LightCycler (Roche Diagnostics, Indianapolis, IN, USA). The relative expression level for each candidate gene was calculated using the 2^–ΔΔCT^ method with *LeEF-1α* as an internal reference gene. The primers used for qRT-PCR reactions are listed in the Additional file 12.

### Availability of supporting data

The data sets supporting the results of this article are included within the article and its additional files.

## Additional files

Additional file 1:
**List of the predicted** ***SlbHLHs*** **and their corresponding information.**
Additional file 2:
**The distribution ratio of** ***SlbHLH*** **genes on chromosome of tomato.**
Additional file 3:
**The duplication of** ***SlbHLHs*** **with low- and high-stringency.**
Additional file 4:
**Segmental duplication analysis for** ***SlbHLH*** **genes in whole tomato genome.**
Additional file 5:
**Segmental duplication and gene alignment.**
Additional file 6:
**Intron distribution in the coding sequence of the bHLH domain of** ***SlbHLH*** **genes.**
Additional file 7:
**Neighbor-joining phylogenetic tree based on the bHLH domains of AtbHLHs and SlbHLHs.**
Additional file 8:
**Summary of the predicted functions of SlbHLHs by comparative analysis with AtbHLHs.**
Additional file 9:
**RPKM values of expression of** ***SlbHLHs*** **in ten tissues of tomato.**
Additional file 10:
**Maximum likelihood tree of plant bHLH domains.** This is a detailed representation of the tree shown in Figure 7. Additional file 11:
**The bHLH protein sequences of tomato and other species.**
Additional file 12:
**Primers used for qRT-PCR analysis.**
