# Supplementary material for: Genome-wide identification and characterization of the bHLH gene family in tomato
Source: BMC Genomics. 2015 Jan 22;16(1):9. doi: 10.1186/s12864-014-1209-2 (PMC4312455; doi:10.1186/s12864-014-1209-2)
Supplement: Additional file 6: — Intron distribution in the coding sequence of the bHLH domain of SlbHLH genes. [file 12864_2014_1209_MOESM6_ESM.pdf]

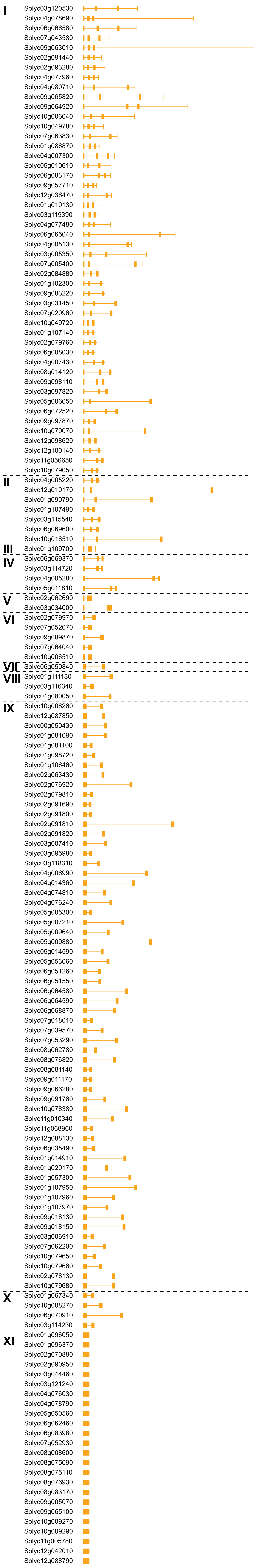


**Additional file 6. Intron distribution in the coding sequence of the bHLH domain of SlbHLH genes**
The 11 different distribution patterns, which designated I to XI are named by the Figure 3. The blocks (solid rectangles) are exon regions, and the thin lines are intron regions in the coding sequence of the bHLH domain for each gene.
