# Supplementary material for: Genome-wide identification and characterization of the bHLH gene family in tomato
Source: BMC Genomics. 2015 Jan 22;16(1):9. doi: 10.1186/s12864-014-1209-2 (PMC4312455; doi:10.1186/s12864-014-1209-2)
Supplement: Additional file 5: — Segmental duplication and gene alignment. [file 12864_2014_1209_MOESM5_ESM.pdf]

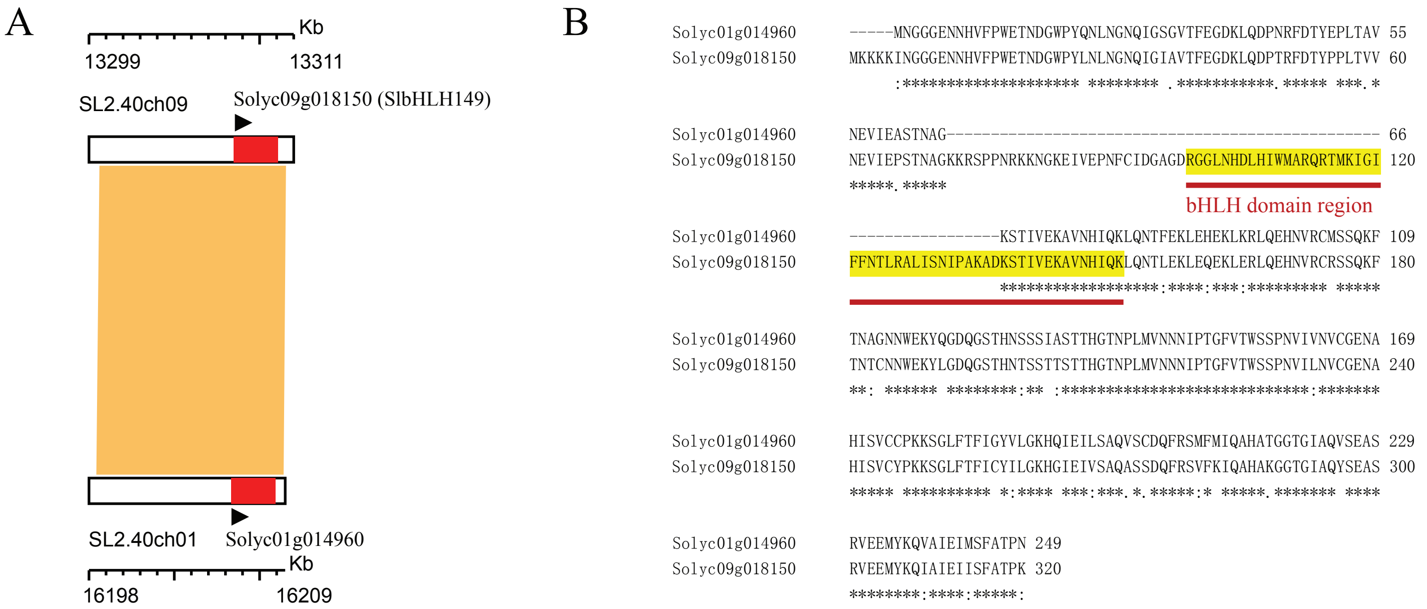

Additional file 5. Segmental duplication and gene alignment

Orange block color indicates a segment duplicated match, while red blocks indicate genes in the segmental duplication region. A. One of the segmental duplications on the chromosome between Chr.9 and Chr.1. The length of segmental duplication is more than 10 kb. B. Alignment between Solyc09g018150 (SlbHLH149) and Solyc01g014960. The yellow color and red line represent the bHLH domain region in the SlbHLH149. The gaps are deletion in the Solyc01g014960 compared with SlbHLH149.
