# Supplementary material for: Genome-wide identification and characterization of the bHLH gene family in tomato
Source: BMC Genomics. 2015 Jan 22;16(1):9. doi: 10.1186/s12864-014-1209-2 (PMC4312455; doi:10.1186/s12864-014-1209-2)
Supplement: Additional file 10: — Maximum likelihood tree of plant bHLH domains. This is a detailed representation of the tree shown in Figure 7. [file 12864_2014_1209_MOESM10_ESM.pdf]

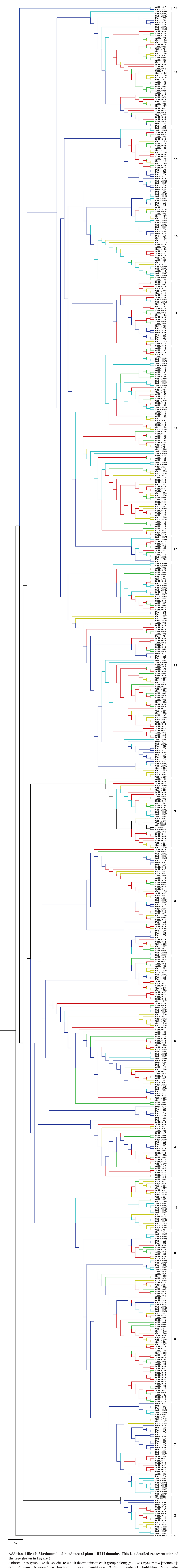

Additional file 10. Maximum likelihood tree of plant bHLH domains. This is a detailed representation of the tree shown in Figure 7

Colored lines symbolize the species to which the proteins in each group belong (yellow: *Oryza sativa* [monocot]; red: *Solanum lycopersicum* [eudicot]; green: *Arabidopsis thaliana* [eudicot]; light-blue: *Selaginella moellendorffii* [lycophyte]; dark-blue: *Physcomitrella patens* [moss]; black: *Volvox carteri*, *Chlamydomonas reinhardtii*, and *Cyanidioschyzon merolae* [chlorophytes and red algae]).
