# Supplementary material for: Genome-wide identification and characterization of the bHLH gene family in tomato
Source: BMC Genomics. 2015 Jan 22;16(1):9. doi: 10.1186/s12864-014-1209-2 (PMC4312455; doi:10.1186/s12864-014-1209-2)
Supplement: Additional file 7: — Neighbor-joining phylogenetic tree based on the bHLH domains of AtbHLHs and SlbHLHs. [file 12864_2014_1209_MOESM7_ESM.pdf]

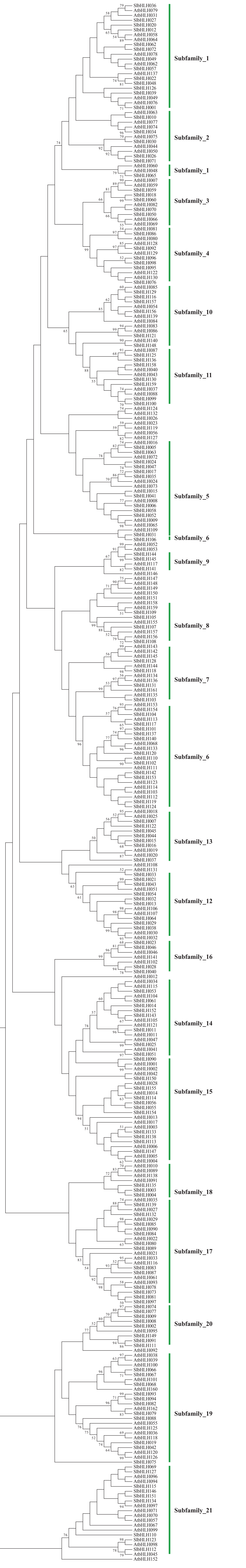

Additional file 7. Neighbor-joining phylogenetic tree based on the bHLH domains of the AtbHLHs and SlbHLHs

The phylogenetic tree constructed using MEGA5. The protein name of *Arabidopsis thaliana* and tomato (*Solanum lycopersicum*) starts with "At" and "Sl", respectively. The bootstrap values are shown for each branch, which is supported by a bootstrap value of > 50% in the phylogenetic clades. The subfamily groups are defined by Figure 4.
